# Attitudes to End-of-Life Care and Voluntary Assisted Dying Amongst Members of the Australian Jewish community

**DOI:** 10.1007/s10943-024-02028-1

**Published:** 2024-03-25

**Authors:** Eli W. Janover, Camille La Brooy, Jennifer Philip, Sophie Lewis, Ian Kerridge, Paul A. Komesaroff

**Affiliations:** 1 School of Public Health and Preventive Medicine, Monash University, Melbourne, 3004 Australia; 2 Melbourne Medical School, University of Melboure, Melboure, Australia; 3 School of Health Sciences, University of Sydney, Sydney, Australia; 4 Sydney Health Ethics, University of Sydney, Sydney, Australia

**Keywords:** Voluntary assisted dying, End-of-life care, Ethics, Jewish community, Essentialism

## Abstract

The implementation of voluntary assisted dying (VAD) in the Australian State of Victoria in 2019 has stimulated discussions about end-of-life care and dying in many communities. Various attempts have been made to represent the attitudes of the Jewish community, a distinct culturally and linguistically diverse (CALD) group, in terms that suggest a unified set of opinions that opposes VAD policies. This research aimed to explore attitudes to VAD in the context of end-of-life care held by members of the Victorian Jewish community. A descriptive qualitative methodological design was employed. Ten Victorians who identify as Jewish were recruited and participated in in-depth, semi-structured interviews. Reflexive thematic analysis was carried out on the transcripts to identify key themes, attitudes and preferences in relation to end-of-life care, death and dying, and VAD. Three themes were identified: “complexity and variation”, “similarities”, and “factors influencing attitudes to VAD and end-of-life care”. A significant degree of diversity was apparent, ranging from highly supportive of VAD to advocacy for a total repeal of the policy. The results indicate that images of how Victorian Jewish individuals feel towards VAD based on essentialised notions about the community and belief systems are not supported by the evidence. In reality, considerable diversity of attitudes exists towards VAD and end-of-life care. We conclude that it is important that policymakers and members of the broader society avoid stereotypes that falsely characterise this specific community and, by implication, other CALD groups, particularly in terms that ignore internal diversity regarding belief systems, social attitudes and ethical perspectives.

## Introduction

Voluntary assisted dying (VAD) came into effect in Australia in June 2019 with the activation of the Victorian Voluntary Assisted Dying Act, 2017 (“the VAD Act”) which had been passed 2 years previously following extensive community consultation (Parliament of Victoria, 2016; Victorian Government Department of Health, 2019; VAD Act). The Act allows patients who have been diagnosed with a terminal disease that ‘is causing suffering… that cannot be relieved in a manner that the person considers tolerable’ to be prescribed a substance that they may take which will end their lives at a time of their choosing (VAD Act). The implementation of this law represented a major shift in the direction of end-of-life care in Australia, which has reverberated through the biomedical, legal, and social spheres, generating vigorous and ongoing discussion.

It is often claimed that Australia is a diverse and multicultural society in which individual cultural groups are embraced and respected, and differences fostered. However, such characterisations often generate stereotypes in which cultural groups are caricatured or “essentialised” (Kowal, 2008). Indeed, it has been claimed that the Australian model of multiculturalism creates a tension between two diverging impulses: a desire to eliminate inequality and an attempt to preserve difference (Kowal, 2008).

The Jewish community is one of Australia’s oldest communities, and it plays an important role in cultural and political dialogues. Whilst it is itself culturally diverse (Graham & Markus, 2018), it is often assumed that its members may hold a view of the practice of VAD and other end of life issues that are more conservative than that of the rest of the population. The Institute for Judaism and Civilisation and the Rabbinical Council of Victoria, both religiously orthodox-oriented organisations, presented submissions to the Victorian Government’s Inquiry into End-of-Life Choices in 2016. Although prepared by a small group of people in the absence of any process of community consultation, these submissions claimed to represent both the views of all Jewish people and an authoritative Jewish position on VAD. They argued that Judaism opposes active voluntary euthanasia and implored the government to withdraw the VAD bill (Rabbinical Council of Victoria, 2017; Standing Committee on Legal & Social Issues, 2015; Cowen, 2015).

Media reports are also occasionally cited as evidence that Jews are averse to VAD. For example, an article published in the widely read on-line magazine *The Conversation* presented the purported view of Jewish law towards VAD and suicide, concluding that it was the Jewish view that greater emphasis should be placed on education campaigns to strengthen the value of life and its “fundamentally sacred nature” (Sinclair, 2017). An article published by the Australian Broadcasting Corporation (ABC) presented the view of an Orthodox rabbi in Perth, who averred that his community was implacably opposed to VAD (Shine, 2019), even claiming further that, the entire “Hebrew, Islam and Catholic faiths are… opposed to” VAD (Shine, 2019). In October 2017, religious leaders from the Sikh, Islamic, Jewish, Christian, Buddhist and Hindu communities united to release a joint statement on the steps of the Victorian Parliament which urged the government to reject the proposed VAD legislation (Musse et al. 2017).

These statements, submissions and articles may have contributed to an image of Victoria’s Jewish community as a homogenous, monolithic social group whose members share identical attitudes towards VAD and end-of-life care issues in the eyes of policymakers and the wider society. However, there is evidence that this community is in fact quite diverse and that within it a great range of attitudes exists on the matters of end-of-life care, although relatively little is known about their view about VAD in particular (Grinberg et al., 2018; Gesundheit et al., 2006) and some Rabbinical authorities have made declarations clearly at odds with the idea that all Jews are against VAD (Romain & Carey, 2021).

It is the aim of this paper to examine the variety of views held by Jewish people in Victoria and to subject to scrutiny the premise that that these can be characterised uniformly or as representing a single agreed position. In doing so, we are concerned not with theological questions about how VAD might be understood in the light of scholarly texts but only with the actual views held by Jewish people in the Victorian community.

### Australian Jews

The Australian Jewish population is estimated to be 113,000, making up approximately 0.5% of the national population (Graham & Markus, 2018). The community is well established and was largely built out of several waves of immigration throughout the twentieth century. Currently, the largest Jewish communities in Australia consist of numerous synagogues, congregations, communal organisations, and interest groups, mainly concentrated in Victoria and New South Wales (Graham & Markus, 2018; Jewish Community Council of Victoria, 2021; New South Wales Jewish Board of Deputies 2022). The community is diverse, with Australian Jews affiliating with Judaism in varying ways, as shown in Table 1.

**Table 1:** Streams of Judaism and estimated proportion, Australia, 2017

Self-identified stream of judaism affiliation	Estimated Jewish population proportion %
Strictly Orthodox/Haredi	4
Modern Orthodox	18
Traditional	30
Conservative (Masorti)	3
Progressive	11
No-denomination, Just Jewish	12
Secular	21
Mixed religion (Jewish and another religion)	1

Adapted from Graham & Markus, 2018, p13) (Graham & Markus, 2018)

### “Essentialism” and Culturally and Linguistically Diverse Communities

Whilst it is important to find ways of representing in general terms the values, preferences and needs of different cultural groups, it is also important to respect the variety of views that may be held in those communities. This presents a challenge to find a theoretical framework that avoids the extremes both of “essentialism”—which assumes unanimity of outlook—on the one hand, and “anti-essentialism”—the denial of common themes or shared understandings altogether—on the other. In this project, we sought to establish the outline for such a framework to capture the array of attitudes and beliefs of Jewish people in Australia towards VAD.

In its most general usage, essentialism is the view that it is possible to specify the nature of a collection of objects or things in terms of supposedly inherent or fundamental attributes. Applied to social categories, this implies that different groups—defined in terms of religion, gender, cultural background, race, or other features—each have their own abiding characteristics, or “essences”, which determine their forms, structures, internal dynamics, and relationships with other groups. Some scholars (Nathan, 2015; Sen, 2007) suggest that essentialism implies a link between social categories and “human nature”, whereas others (McSweeny, 2009) may postulate other causal origins. Whatever the case, all forms of essentialism assume that social groups are static, homogeneous, deterministic, and bounded.

Even where an underlying philosophical commitment to essentialist principles is not explicitly declared, it is common in everyday discourse for certain social groups to be described informally in essentialising terms. For example, it is often assumed that members of racial groups or religious communities, or those occupying gender roles—share common characteristics that are unchanging (Diesendruck & Menahem, 2015) and allow extensive additional inferences to be drawn about them. By contrast, non-essentialism or anti-essentialism challenges these notions, claiming that common beliefs, attitudes, or other shared properties are contingent and vary according to historical and social or cultural conditions. Such a claim may even be made for “human nature” itself. Consequently, non-essentialism, in opposition to essentialism, posits that social groups hold attributes that are dynamic, fluid, heterogeneous, changeable, and have blurred boundaries (Nathan, 2015).

Essentialism and anti-essentialism have both strengths and weaknesses. The former can support common identities and the development of a sense of solidarity but can also support stereotyping of individuals, constricting their behaviours, beliefs, and attitudes to ideas or images associated with their respective cultures (Nathan, 2015; Holliday, 2010; Osland et al., 2000). The latter can acknowledge the richness of diversity and difference but deny common interests and needs and obscure systemic causes of inequity and discrimination and entrenched disparities of power and privilege in favour of an atomised, individualistic view of social processes.

The existence of essentialised perceptions, in healthcare fields (and beyond), of “Culturally and Linguistically Diverse” (CALD) communities and minority groups, including Jewish communities, is well documented in the scientific literature (Adusei-Asante & Adibi, 2018; Curtis et al., 2019; Diesendruck, 2013; Downing & Kowal, 2011; Jennings et al., 2018; Owens & Randhawa, 2004; Truong et al., 2014). Additionally, it is common for such communities to be essentialised within and by the scientific literature itself (Baker et al., 2016; Adusei-Asante & Adibi, 2018; Beagan, 2015; Dawson et al., 2022; Martínez Mateo et al., 2013). On the other hand, anti-essentialist viewpoints have been used to argue against policies that support affirmative action or attempts to overcome systemic causes of inequities in wealth, educational opportunity or health status.

As indicated above, Jewish groups have often been essentialised, in the health literature and the media, by policymakers and in policy development processes in Australia, specifically in discourses surrounding assisted dying laws and other end of life (EOL) care practices such as palliative or hospice care (Hiruy & Mwanri, 2014; Falk, 1998). Such assumptions have been applied to debates about voluntary assisted dying and EOL care, potentially in a manner that limits the possibility for diverse voices to be heard. A potential consequence of this, is that it restricts the ability of health and social policies to address the needs of the entire populations they purport to serve.

To help find a way through this conundrum, this study set out to identify the range of attitudes held by Victorian Jews on the topics of EOL care and VAD. It also sought to examine how those attitudes were formed in relation to religious, cultural and other influences and to assess the extent of any relative homogeneity or heterogeneity of attitudes in this community. In this manner, we sought to characterise the extent of the diversity of attitudes held by Victorian Jews towards EOL care, death, and VAD.

To achieve these objectives, we adopted a descriptive qualitative methodological study design. The data were contextualised within the current literature on the topic of attitudes to VAD and EOL care more generally in relation to the ideal types of essentialism and non-essentialism.

## Methods

This study was undertaken as part of a larger project that assessed the impact and consequences of the Victorian Voluntary Assisted Dying Act. We employed a qualitative approach well suited to in-depth exploration of participants’ diverse perspectives on EOL care and VAD. The study received ethics approval from the Monash University Human Research Ethics Committee (Project Number: 19884) and all participants provided written informed consent.

Participants were sampled purposively, in relation to the following characteristics: experience, occupation, age, gender, Jewish affiliation, connection to Jewish community, and religious observance. Participants were purposively sampled, using community recruitment strategies. People interested in taking part in the study were identified via community advertisements and personal contacts. Individuals over the age of 18 years who lived in the Australian state of Victoria and who self-identified as Jewish were included. Exclusion criteria included a diagnosis of a terminal illness to exclude situational reactions in contrast to considered attitudes and beliefs.

A total of ten individuals participated in the study. After providing informed consent, two data collection methods were utilised: a demographic questionnaire and semi-structured interviews. The demographic questionnaire collected participants’ age bracket, gender, self-reported ethnicity and religious affiliation, and gathered a pre-interview indication of the extent to which they supported or did not support the practice of VAD—via a slider tool. Semi-structured interviews were conducted using a videoconferencing facility and recorded between May and August 2022.

Interviews were conducted and recorded via “Zoom” and ranged between 30 and 60 min in duration. Each interview followed a general structure outlined in an interview guide, which was modelled on published guides—specifically the work of Zamer and Volker (2013) and Bonura et al. (2001)—and adapted to local needs. Where participants lacked pertinent knowledge of the VAD Act, the interviewer provided a brief explanation, taking care to avoid suggestive or loaded language favouring one view or another. Interviews were transcribed verbatim using the transcription software “Otter.AI”, and further edited manually by two of the authors. Identifying information such as names was then removed to protect anonymity of each participant.

The interview data were analysed thematically and inductively. “NVivo” software was used to organise and code data. One of the authors initially analysed the transcripts guided by Braun and Clarke’s reflexive thematic analysis (TA) approach (Braun & Clarke, 2021, 2022). The first author who conducted the interviews and the analysis identifies as Jewish, indicating potential researcher positionality which he sought to address by reflective conversations with the other investigators and the conscious adoption of an open and disinterested disposition in the conduct of the interviews, the analysis of the data and the composition of the manuscript.

## Results

### Participant Characteristics

Participant characteristics are summarised in Table 2. Age brackets ranged from under the age of 25 up to over the age of 61. Forty percent were female. Substantial diversity of connection to Jewish identity and Judaism was observed amongst the participants, including in the areas of: ethnicity; religion and religious affiliation; belief and non-belief; and community and cultural connections. Two participants identified with Cultural/Secular Judaism, two identified with traditional Judaism, two identified with Progressive Judaism, two identified with Orthodox/Modern Orthodox Judaism, and two identified with Ultra-Orthodox Hasidic Judaism. Participants reported varying levels of knowledge of the VAD Act, and all reported having known a person or persons who had had a terminal illness. Participants were diverse in their beliefs about God, including atheists, those who believe in “something greater than themselves”, and those who believe in a “Jewish God”. They also expressed diverse connections to their Jewishness and to the local Jewish community.

**Table 2:** Participant characteristics

Participant	Gender and age bracket (pre-interview form response)	Religion (pre-interview form response)	Ethnicity (pre-interview form response)	Religious affiliation (Interview response) & abbreviation in parentheses	Birthplace and generation Australian (Interview response)	Occupation/field (Interview response)	Highest level of Education (Interview response)	Communal involvement (current and/or past) (Interview response)
Participant 1	F 31–40	Judaism	Caucasian	Progressive Judaism (P)	Australia (rural)—6th generation	Health promotion	Postgraduate	Multiple Progressive Jewish communal organisations, youth groups, community aged care
Participant 2	M < 25	Jewish	Ashkenazi	Orthodox Judaism (O)	Australia—2nd generation	Student	Undergraduate (current student)	Community journalism, youth groups
Participant 3	F 51–60	Jewish	South African/Australian/Jewish	Traditional Judaism (T)	South Africa—1st generation	Finance/law	Undergraduate	Community religious services, Jewish learning, Cultural organisations,
Participant 4	F 51–60	Not religious	Jewish	Cultural Judaism (C) (agnostic)	Israel—1st generation	Academia/education	Postgraduate	General engagement
Participant 5	M > 61	Jewish	Jewish	Cultural Judaism (C) (atheist)	Australia—2nd generation	Retired/real estate	Undergraduate	General engagement
Participant 6	M 26–30	Jewish	Jewish/Israeli/Iraqi/Polish	Orthodox Judaism (O)	Australia—3rd/2nd generation	Medicine and religious services	Postgraduate	Youth groups, Jewish learning, community disability services, social justice organisation, community religious services
Participant 7	M > 61	Jewish	Caucasian	Traditional Judaism (T)	Australia—2nd generation	Medicine	Postgraduate	Community ambulance service, community medical organisation, youth groups
Participant 8	M > 61	Jewish	European	Chabad, Ultra-Orthodox Judaism (UO)	Australia—2nd generation	Medicine and religious services	Postgraduate	Community religious services
Participant 9	M 31–40	Jewish	Yemenite	Chabad, Ultra-Orthodox Judaism (UO)	Israel – 1st generation	Religious services	High school	Community religious services
Participant 10	F < 25	Jewish	Caucasian/Jewish	Progressive Judaism (P) (agnostic)	Australia—2nd generation	Student	Undergraduate (current student)	Youth groups

**Fig. 1:**
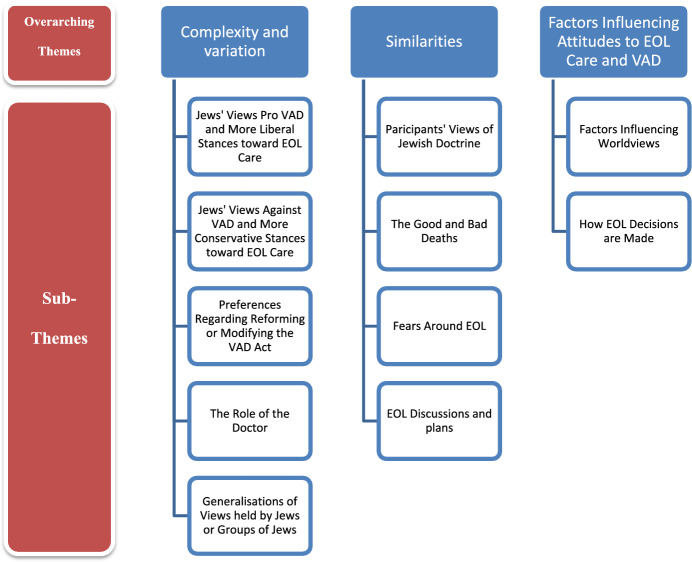
Overarching Themes and Sub-Themes

Analysis of the qualitative data generated three overarching themes: “Complexity and Variation amongst Participant Attitudes Towards Voluntary Assisted Dying and End-of-Life Care”, “Similarities in Participant Attitudes Towards Voluntary Assisted Dying and End-of-Life Care”, and “Support for and Criticisms of Essentialising Discourses”. The themes were divided into several subthemes, as displayed in Fig. 1. These will be described in turn.

### Theme 1: Complexity and Variation amongst Participant Attitudes Towards Voluntary Assisted Dying and End-of-Life Care

Throughout the interviews a significant level of complexity and variation was present about VAD and EOL care. Participants presented nuanced attitudes ranging from those who would like the VAD Act to be repealed to those who would like to see VAD made more easily accessible. Other subthemes included participant attitudes regarding the role of doctors in EOL care and participant preferences regarding reforming or modifying the VAD Act. Amongst these subthemes, significant complexity and variation were observed across the participants.

Several participants were supportive of VAD because they felt that it provided greater autonomy and dignity to vulnerable individuals in their time of need:

I think [VAD is] great… I think it’s the ultimate in human dignity to make choices about how you live and how you die. (Participant 1, F, 31-40, P)^1^

One participant who was supportive of VAD explained that her worldview is shaped by secularism and evidence, in contrast to one that is shaped by religious authority:

Probably my secular worldview. If I wasn’t secular, then I might have taken for granted what is given to me by the rabbis … I don’t take information from authorities, we tend to take from evidence as much as we can … freedom of thought, freedom of decision, these values come from this kind of worldview. (Participant 4, F, >61, C)

The same participant went on to explain how she would like to see a broadening of access to VAD and that the current VAD system is too limited:

It gives kind of very limited options when it’s pretty clear that you only have a year to die or something like that. So, it’s limited to a very small portion of the population that kind of falls into these very strict rules. Probably I would have liked to see it broadening a bit. (Participant 4, F, >61, C)

Other participants viewed VAD as a humanitarian EOL choice. When discussing his experience as a volunteer with a VAD advocacy organisation, participant 5 felt privileged to be able to aid someone in reaching their desired EOL goals as a witness by attesting to the individual’s capacity in the VAD process:

I felt uniquely privileged to be able to assist this woman achieve a real humanitarian goal of control of her own life and her own death. (Participant 5, M, >61, C)

In contrast, other participants were against VAD due to their religion and adherence to Jewish law. Participant 9 believed strongly in the sanctity of life:

Life is holy, and life belongs to Hashem (God). We’re not allowed to end it any earlier. (Participant 9, M, 31-40, UO)

This participant expressed the view that the VAD Act should be totally repealed:

If I was the king of Victoria, I would immediately repeal [the VAD Act]… I would want everywhere in the world for these kinds of laws to be repealed. And for there to be the true safeguards, which is to safeguard every life from voluntary assisted dying. (Participant 9, M, 31-40, UO)

Participant 8 believed that VAD is already a step too far down “the slippery slope”:

I don’t even think of it as a slippery slope. You know, like because it’s not a slippery slope. It’s just a cliff. Once you allow that, then [it has gone too far]. (Participant 8, M, >61, UO)

However, participant 7 sought to refute this argument. As the son of Holocaust survivors, he believed that slippery slope arguments connected to Nazi eugenics were bogus. To the contrary, he believed that his parents’ experiences of suffering in the Holocaust caused them to instil in his generation the value of the alleviation of the suffering of others:

There are places all over the world where voluntary assisted dying has been introduced, and we haven’t seen that slippery slope, and it’s not like Nazi eugenics… [VAD] is all about preserving someone’s autonomy. Eugenics was all about destroying that autonomy… [My parents] saw a lot of people suffering in the camps. And I think that that’s probably something that’s been transferred from that generation to my generation about trying to alleviate suffering. (Participant 7, M, >61, T)

Further complexity emerged in the discussions about the “separation of Church and State”. Participant 2 reported a tension between his religious beliefs and his belief in personal liberties:

[For] the question of, “…is voluntary assisted dying immoral?” …if I had to give an answer, [it] would be informed on Halacha. But at the same time, that shouldn’t interfere with like individual liberties…I think personal liberty is very important… My religious beliefs shouldn’t have any effect on what some random person living like on the other side of the country does in their own time… But then again, there still does have to be some sort of limit on it. (Participant 2, M, <25, O)

Participant 6 agreed with the separation of religion and state and elaborated on his desire to see more religious “soft” freedom in society:

I don’t want religious values necessarily imposed on a secular society… I believe in separation of those two things. And I believe that the flip side of that is people need to be given the freedoms to practise their religion, not just in terms of like, the hard freedoms… but also in terms of like the cultural acceptability, and the more softer side of what being free to practice your religion openly looks like. (Participant 6, M, 26-30, O)

### Theme 2: Similarities in Participant Attitudes Towards Voluntary Assisted Dying and End-of-Life Care

Although significant variation was observed amongst participant attitudes towards VAD and EOL care, there were also several notable areas of similarity. Subthemes included participant perceptions of “good deaths” and “bad deaths”, fears around EOL, and EOL discussions and planning, as well as participant perceptions of Jewish doctrine. Similarities in these areas were observed across a majority of the participants.

Participants largely held similar fears about EOL, of gradual neurodegeneration, of prolonged suffering, and of a loss of agency:

My fear, I guess, is around having the option or the choice [to end my own life], whether that’s through voluntary assisted dying, or by my own hand, I would feel fearful at the idea of that not being available to me. (Participant 1, F, 31-40, P)

I wouldn’t want to find myself living 15 years with dementia. (Participant 4, F, 51-60, C)

Fear of dementia and neurodegeneration in old age was a common theme. Many participants reported experiences with loved ones or acquaintances who had had such conditions. Several participants hoped that the VAD law could be made more accessible to individuals and families experiencing debilitative neurodegenerative diseases.

Similarities were also observed amongst descriptions of “the good death” and what this might entail. By and large, participants described similar characteristics, such as ideas of peacefulness, dignity, a lack of prolonged suffering, dying after a life well lived, dying in one’s sleep, dying at home surrounded by family, and where the person has some control or choice over their EOL:

I think a good death is one that you have the ability to influence or to make decisions in and around or have control over as much as possible, where your dignity… is upheld, where your choices are respected. And ideally with a minimal amount of pain and suffering… The ideal what we’d all like is at the end of a long and fulfilling life to just drift away in our asleep with no knowledge of otherwise. That sounds like a pretty good thing to me (Participant 1, F, 31-40, P).

It’s about there not necessarily being too much suffering there. I prefer at home, rather than in a hospital with a lot of beeping machines. Yeah, being given the time in the lead-up to the death to have the important conversations that someone would want to have with their family or loved ones or make their peace with God… First of all, I’d like to live before I die… I’d really like to have a good innings … I think that makes death a bit easier. If you know that you’ve done your best during your life. [I’d like to die] at home surrounded by family and friends. (Participant 6, M, 26-30, O)

Participant 9 directly referred to Jewish religious texts and traditions when describing his idea of a “beautiful death”:

The good death will be like the death of Aaron, the brother of Moses... [The Rashi commentary] on the Torah, says that Moshe [Moses] desired that death because it was such a beautiful death... Because it was like, go up to the mountain, lie down on the bed... and now God takes your soul like, it’s called “dying with a kiss of God like Aaron”. Meaning if a person dies in his sleep, that’s a beautiful death, meaning if a person dies in ripe old age, having children and grandchildren with no pain and no illness... that would be the beautiful death. (Participant 9, M, 31-40 UO)

Whilst all participants shared views about the importance of a good death as a death where suffering is minimal, they diverged in their views about whether VAD was a means of achieving such a death. For example, some participants, in spite of agreeing that they desired deaths with little suffering, did not accept that ‘choice’ about how you die could make a good death possible:

[Its] not something that someone could choose. You know, “I’m 60 and I’m sick of living, so I’m just going to take some cyanide before I go to bed”. Nup, I don’t think so. That’s not a good death. (Participant 8, M, >61, UO)

Participants differed on what should be considered “positive” and “negative” features of the dying process. For example, Participant 9 described a scene of active voluntary euthanasia containing many of the characteristics that other participants attributed to a “good death” which he nonetheless considered would be a “bad death”:

I remember one time seeing ...a video ...of somebody in the United Kingdom who was just diagnosed with [a] very advanced form of multiple sclerosis... He didn’t look extremely ill in the video. And then he sits there in... his house. And he’s listening to Beethoven’s last symphony, and they injected him... the doctor is helping him inject something in his arm. And there he goes on. His wife is by his side, holding his hand. That was one of the most horrifying videos I’ve ever seen in my life. Somebody consciously ending their life. (Participant 9, M, 31-40, UO)

In addition, whilst most of the participants included “choice at EOL” as a part of a good death, this view was not shared by Orthodox participants alone, even though the latter still agreed that VAD should not necessarily be outlawed as a result of religious beliefs.

### Theme 3: Support For and Criticisms of Essentialising Discourses

In response to questioning about how they saw “a Jewish view” of VAD, a vigorous array of responses was provided.

Participants often supported the view that a “Jewish opinion” on VAD existed or asserted that Jewish individuals would adopt a certain attitude towards VAD and EOL care based on their culture or beliefs. Participants often stated that the opinion of Judaism or the “Jewish view” towards VAD and EOL care was quite conservative, even when their own attitudes departed from this characterisation:

My understanding is that generally speaking, Judaism is quite conservative on this issue. (Participant 2, M, <25, O)

Several participants divided the Jewish community into their religious streams and then generalised how each stream may feel towards VAD:

You can kind of like simplify it per stream [of Judaism]… Generalising—Progressive Jews would be for it, I believe, in certain circumstances. When you get anything other than Progressive, so Modern Orthodox, Orthodox, etc, when you go up the chain, essentially no [support for any form of assisted dying]. (Participant 10, F, <25, P)

Participants often expressed somewhat caricatured interpretations of Jewish religious doctrinal views towards VAD other than their own. Remarkably, the stated views of participants frequently did not align with their own descriptions of the presumed attitudes held by those following what they regarded as Jewish doctrinal views towards VAD.

The concept of *pikuach nefesh* is “to save a life” and it’s a principle of Judaism. But to me, there’s definitions of life. And if I was to insert …a couple of words it would be “to save a life with dignity”. So, I don’t think that we need to uphold life above everything else when that life is not a life worth living. (Participant 7, M, >61, T)

Even whilst seeking to characterise a general “Jewish view” of VAD several participants expressed hesitation about whether a singular Jewish view actually existed; as Participant 2 discussed, Jewish communities, like all communities have a multiplicity of views on social issues. Thus, it was necessary to consider the specificities of the person in relation to VAD:

Take any community and put like one issue, and you’ll have a whole spectrum of views. So I don’t see why it would be any different about this… I don’t think the community will have a perspective, I think it really depends on each individual. (Participant 2, M, <25, O)

Similarly Participant 6 cautioned against claims of universalising discourse on VAD, suggesting that assumptions such as a singular view that privileges life over quality of life sidelined the views of the many within the Jewish community who do not ascribe to such views:

Any time someone says, you know, “Judaism says...”, you probably should stop listening at that point. Because the reality is… it’s hard to talk in sort of any unified way… But…I’d say, there’s certainly a privileging of life and a privileging of even short amounts of life without an overarching focus on the quality of that life. And with a sort of aversion towards doing active things to take away that life. (Participant 6, M, 26-30, O)

Notably, however, Orthodox Jews were more likely to believe in a single Jewish view than those of non-orthodox affiliations:

Jewish people are not supposed to kill themselves. (Participant 8, M, > 61, UO)

…all people who are Orthodox religious, will understand the sanctity of life, and will not be fiddling around with, “ohh maybe his life’s not worth living”. No. We don’t do that. (Participant 8, M, >61, UO)

In all situations, the Torah says that you can’t do the voluntary assisted dying... The Jewish perspective is Torah (Participant 9, M, 31-40, UO).

However, this belief seemed often to be based on an understanding of a universal, undeniable truth, where all of humanity is, or should be, subject to religious law:

…one of the seven Noahide laws includes the prohibition against murder, which means it’s not just for Jewish people, but for all people in the world. There’s many details in each one of these seven laws, they have a lot a lot of sub-detail. And in the category of murder, the whole subject of euthanasia, abortion, and other forms of murder are addressed and that’s why it’s very, very important for me. (Participant 9, M, 31-40, UO)

The role and the job of a Jewish person in the world is to be Hashem’s (God’s) messenger, to reveal godliness, not just [to] fellow Jews, but also [to] all of humanity… [Jews] have to be there in the world, revealing godly light in... And the way to do that is through the Torah and mitzvot (good deeds/religious obligations) … A Jew’s connection with God is essential because of his actual, like existence, his purpose. (Participant 9, M, 31-40, UO)

## Discussion

Australia is a highly diverse multicultural nation (Boese & Phillips, 2011) in which researchers, policymakers, and the broader society need to balance varying attitudes, beliefs and value perspective of different communities and cultural groups.

The Jewish community is a distinct CALD community in Victoria that is often assumed to share common views about key social and ethical issues, in respect of which a picture is often presented of a homogeneous group of individuals characterised by fixed views that are presumed to be constitutive of its shared identity. As previously noted, such an assumption has played an important role in the public debates leading up to the introduction of VAD in Victoria, with multiple submissions to parliament (Cowen, 2015; Standing Committee on Legal & Social Issues, 2015), position statements (Rabbinical Council of Victoria, 2017) and media articles (Shine, 2019; Sinclair, 2017) purporting to present a unified, authoritative ‘Jewish view’ towards EOL topics and VAD.

The present study has shown that such essentialised views of the Jewish community are largely unfounded. Rather, despite some shared attitudes and opinions, mainly relating to high-level concepts about images of what constitutes the good life, there is a wide variation in views about the key substantive issues surrounding EOL care and VAD. Paradoxically, even in the face of their own heterodox perspectives, individual Jews themselves often perpetuate the image of a unified, doctrinal order that inherently commands universal agreement.

In our interviews with individuals from all the major Jewish cultural and religious affiliations in Australia, views expressed about VAD ranged from liberal and permissive to conservative and critical. There was similar variation in attitudes to the need for reform or modification of the VAD Act, including a desire for no change, further liberalisation, additional limits, and total repeal. Opponents of VAD were more likely to adhere to the Ultra-Orthodox or Orthodox streams of Judaism, and Progressive or less religious Jews were more likely to be supportive. These findings are consistent with those of previous studies, such as those by Chakraborty et al. (2017), Baeke et al. (2011b), and Wenger and Carmel (2004), which found that more liberal views towards EOL care and VAD are negatively correlated with higher levels of observance of religion and Orthodox Judaism, whilst recognising that, as Baeke et al.(2011b) and Schweda et al. (2017) found, attitudes are not always perfectly matched with participants’ religious or cultural identities.

Interestingly, even in relation to the event that most vividly defines the self-identity of Australian Jewish people, the Holocaust, participants adopted seemingly contrasting views and arguments. These included both the assertion that the experience of the Holocaust inevitably means that VAD must be regarded as a moral affront, and the conclusion no relationship exists between VAD and the Nazi program of eugenics. This debate within Jewish groups is also consistent with the literature. Jewish or Holocaust survivor participants in Schweda et al. (2017), Leichtentritt and Rettig (1999), and Wenger and Carmel (2004) rejected the idea of a “slippery slope” and the comparison between the actions of the Nazi regime and VAD or similar policies, whereas Baeke et al. (2011b) cite Jewish participants who invoke the comparison to the actions of the Nazi regime as a cogent argument against VAD and similar policies.

In the face of these differences, there were some striking points of agreement. These were often at a high level of abstraction, such as the notion of what constitutes a “good death”. Here, participants often referred to common values, such as choice, dignity, and control at EOL; dying in old age; leaving a legacy; dying without suffering; and dying at home surrounded by loved ones. These shared values often transcended religious affiliations and level of religiosity. The existence of a common perception of the “good death” was reported by Leichtentritt and Rettig (2000) who, in addition to the above list, also included the desire to avoid physical pain and suffering, to die at an appropriate age, to leave a legacy, and not to be alone.

Most participants acknowledged the existence of a wide range of attitudes about EOL care and VAD held by Jews or groups of Jews, with several expressing the belief that this variety was similar to that found in the rest of the Australian population. However, remarkably, many at the same time made broad sweeping statements about the Jewish community—for example, about the presumed attitudes of the majority towards VAD. A frequently stated belief, amongst participants of varying religious affiliations, ages, genders and life experiences, was that the doctrines of Judaism opposed VAD and would lead to a conservative attitude towards it. This perception linked the acknowledged Jewish view on the sanctity of life and its prohibition of murder to a judgement about VAD. This view persisted even in spite of the manifestly discrepant views of many of those proclaiming it.

A number of participants divided the Jewish population into religious streams and speculated about the likely views of a majority of each of them. Whilst there is some evidence of a link between interpretations of the Jewish texts and conclusions about EOL decisions and VAD (Baeke et al., 2011a), it is not possible to conclude from our data, or indeed from the published literature, how valid these generalisations are likely to be.

Whilst surprising, the phenomenon of a conflict between personal views and a view about doctrines said to be foundational to one’s belief system is not unprecedented. According to Oyserman (2019), in making sense of their beliefs, people prefer to act in ways that are congruent with their identity, “who they are and who they might become”. It is possible that, after having discussed their Jewish identity in-depth, participants’ felt compelled to articulate their high-level beliefs in identity-congruent ways (Oyserman, 2019), whilst simultaneously, in relation to the particular, concrete case of VAD and EOL care was able to suspend some of these precepts in favour of values, such as autonomy and humanism, that enjoy a privileged status in the wider society. It is possible that secularising trends within elements of the Jewish Victorian population allow some Jewish individuals to distance themselves from traditional Jewish authority and dogma (Graham & Markus, 2018) whilst at the same time, maintaining a sense of respect for and allegiance to it.

This apparent paradox of a rich diversity of views and attitudes and a common self-identify that assumes a process of essentialisation that is manifestly contrary to the empirical reality, has implications for the impact of VAD on Jewish people in the state of Victoria. These may include misleading representations of the views of the Jewish community in public debates and, presumably, confusion amongst many Jewish people themselves. The Victorian VAD law makes provision for conscientious objection amongst health professionals who feel personally unable to participate in VAD. It is possible that for both Jewish practitioners and patients the presence of an inner conflict between personally held beliefs and supposed official doctrines may exacerbate uncertainty and distress at a difficult time.

## Limitations

Our study has both strengths and limitations. It took place immediately after the implementation of the Victorian VAD legislation, thereby providing a reflection of a unique, intense active debate in the community and it included the views of representatives of all the major affiliations within the Victorian Jewish community. However, individuals with a terminal illness were excluded because of concern for ethical complexities associated with their particular vulnerability, and this may have affected the range of views expressed.

The sample size was relatively small, and no claim is made that theoretical saturation has been reached—that is, it is not claimed that the views presented constitute a complete and comprehensive inventory of those held by all Jewish people in the Victorian community. However, the purposive sampling of different streams of Judaism provided sufficient data to establish the existence of a wide diversity of views regarding VAD and EOL care within the Jewish community.

## Conclusion

In conclusion, our study has cast doubt on the common essentialised view of the Victorian Jewish community, at least insofar as attitudes towards EOL care and VAD are concerned. On the contrary, members of the community hold a wide range of views and attitudes towards VAD and EOL care, extending from full acceptance to strong opposition. There are shared views about underlying values, and the concept of a good death, expressed largely in relation to high-level concepts. At the same time, paradoxically, the perception persists, at least amongst some members of the community, that there is a particular and distinctive “Jewish” view of VAD that is held by the entire Jewish community.

These results suggest that neither essentialist nor non-essentialist constructions adequately characterise the views of the Jewish community about VAD and EOL care and that it is important to recognise that whilst it may be possible to provide a general account of views about VAD that are widely shared by many Jews, such generalisations can never do justice to the diversity of attitudes and opinions that exist within the Jewish community.

## Footnotes

1 In the presentation of qualitative data, participant characteristics are referred to in the following order: numerical identifier, gender, age bracket, and religious affiliation.

## References

[CR1] Adusei-Asante, K., & Adibi, H. (2018). The ‘Culturally and Linguistically Diverse’ (CALD) label: A critique using African migrants as exemplar. *Australasian Review of African Studies,**39*(2), 74–94. 10.22160/22035184/ARAS-2018-39-2/74-94

[CR2] Baeke, G., Wils, J. P., & Broeckaert, B. (2011a). ‘There is a time to be born and a time to die’ (Ecclesiastes 3: 2a): Jewish perspectives on euthanasia. *Journal of Religion and Health,**50*, 778–795. 10.1007/s10943-011-9465-9 21253848 10.1007/s10943-011-9465-9 PMC3230754

[CR3] Baeke, G., Wils, J. P., & Broeckaert, B. (2011b). ‘We are (not) the master of our body’: Elderly Jewish women’s attitudes towards euthanasia and assisted suicide. *Ethnicity & Health,**16*(3), 259–278. 10.1080/13557858.2011.573538 21660785 10.1080/13557858.2011.573538

[CR4] Baker, A. E., Procter, N. G., & Ferguson, M. S. (2016). Engaging with culturally and linguistically diverse communities to reduce the impact of depression and anxiety: A narrative review. *Health & Social Care in the Community,**24*(4), 386–398. 10.1111/hsc.12241 25939369 10.1111/hsc.12241

[CR5] Beagan, B. L. (2015). Approaches to culture and diversity: A critical synthesis of occupational therapy literature: Des approches en matière de culture et de diversité: Une synthèse critique de la littérature en ergothérapie. *Canadian Journal of Occupational Therapy,**82*(5), 272–282. 10.1177/0008417414567530 10.1177/0008417414567530 26590226

[CR6] Boese, M., & Phillips, M. (2011). Multiculturalism and social inclusion in Australia. *Journal of Intercultural Studies,**32*(2), 189–197. 10.1080/07256868.2011.547176

[CR7] Bonura, D., Fender, M., Roesler, M., & Pacquiao, D. F. (2001). Culturally congruent end-of-life care for Jewish patients and their families. *Journal of Transcultural Nursing,**12*(3), 211–220. 10.1177/104365960101200305 11989036 10.1177/104365960101200305

[CR8] Braun, V., & Clarke, V. (2021). One size fits all? What counts as quality practice in (reflexive) thematic analysis? *Qualitative Research in Psychology,**18*(3), 328–352. 10.1080/14780887.2020.1769238

[CR9] Braun, V., & Clarke, V. (2022). Conceptual and design thinking for thematic analysis. *Qualitative Psychology,**9*(1), 3. 10.1037/qup0000196

[CR10] Chakraborty, R., El-Jawahri, A. R., Litzow, M. R., Syrjala, K. L., Parnes, A. D., & Hashmi, S. K. (2017). A systematic review of religious beliefs about major end-of-life issues in the five major world religions. *Palliative & Supportive Care,**15*(5), 609–622. 10.1017/S1478951516001061 28901283 10.1017/S1478951516001061 PMC5865598

[CR11] Cowen, S. (2015). Submission 615 - Rabbi Dr Shimon Cowen. [Paper submitted to the Inquiry into end of life choices] Parliament of Victoria. Retrieved from https://www.parliament.vic.gov.au/get-involved/inquiries/inquiry-into-end-of-life-choices/submissions/

[CR13] Curtis, E., Jones, R., Tipene-Leach, D., Walker, C., Loring, B., Paine, S. J., & Reid, P. (2019). Why cultural safety rather than cultural competency is required to achieve health equity: A literature review and recommended definition. *International Journal for Equity in Health,**18*(1), 1–17. 10.1186/s12939-019-1082-3 31727076 10.1186/s12939-019-1082-3 PMC6857221

[CR14] Dawson, J., Laccos-Barrett, K., Hammond, C., & Rumbold, A. (2022). Reflexive practice as an approach to improve healthcare delivery for Indigenous peoples: A systematic critical synthesis and exploration of the cultural safety education literature. *International Journal of Environmental Research and Public Health,**19*(11), 6691. 10.3390/ijerph19116691 35682275 10.3390/ijerph19116691 PMC9180854

[CR15] Diesendruck, G. (2013). Essentialism: The development of a simple, but potentially dangerous, idea. In M. R. Banaji & S. A. Gelman (Eds.), *Navigating the social world: What infants, children, and other species can teach us* (pp. 263–268). Oxford University Press. 10.1093/acprof:oso/9780199890712.003.0048

[CR16] Diesendruck, G., & Menahem, R. (2015). Essentialism promotes children’s inter-ethnic bias. *Frontiers in Psychology,**6*, 1180. 10.3389/fpsyg.2015.01180 26321992 10.3389/fpsyg.2015.01180 PMC4532908

[CR17] Downing, R., & Kowal, E. (2011). A postcolonial analysis of Indigenous cultural awareness training for health workers. *Health Sociology Review,**20*(1), 5–15. 10.5172/hesr.2011.20.1.5

[CR19] Falk, Z. (1998). Jewish perspectives on assisted suicide and euthanasia. *Journal of Law and Religion,**13*(2), 379–384. 10.2307/1051471 15112689

[CR20] Gesundheit, B., Steinberg, A., Glick, S., Or, R., & Jotkovitz, A. (2006). Euthanasia: An overview and the Jewish perspective. *Cancer Investigation,**24*(6), 621–629. 10.1080/07357900600894898 16982468 10.1080/07357900600894898

[CR21] Graham, D., & Markus, A. B. (2018). GEN17 Australian Jewish community survey: Preliminary findings. ACJC Monash University/JCA NSW. Retrieved from https://research.monash.edu/en/publications/gen17-australian-jewish-community-survey-preliminary-findings

[CR22] Grinberg, K., Amzaleg, M., Gamarov-Berman, M., Rubinsky, L., & Itach, S. (2018). Attitudes of the secular and religious Jewish public in Israel to Euthanasia. *Journal of Palliative Medical Care and Research,**1*(1), 1–7.

[CR24] Hiruy, K., & Mwanri, L. (2014). End-of-life experiences and expectations of Africans in Australia: Cultural implications for palliative and hospice care. *Nursing Ethics,**21*(2), 187–197. 10.1177/0969733012475252 23462503 10.1177/0969733012475252

[CR25] Holliday, A. (2010). *Intercultural communication & ideology*. SAGE Publications.

[CR27] Jennings, W., Bond, C., & Hill, P. S. (2018). The power of talk and power in talk: A systematic review of Indigenous narratives of culturally safe healthcare communication. *Australian Journal of Primary Health,**24*(2), 109–115. 10.1071/py17082 29490869 10.1071/PY17082

[CR28] Jewish Community Council of Victoria (2021). Affiliates. Jewish Community Council of Victoria. Retrieved from https://jccv.org.au/jccv-affiliates/

[CR29] Kowal, E. (2008). The politics of the gap: Indigenous Australians, liberal multiculturalism, and the end of the self-determination era. *American Anthropologist,**110*(3), 338–348. 10.1111/j.1548-1433.2008.00043.x

[CR30] Leichtentritt, R. D., & Rettig, K. D. (1999). Meanings and attitudes toward end-of-life preferences in Israel. *Death Studies,**23*(4), 323–358. 10.1080/074811899200993 10558429 10.1080/074811899200993

[CR31] Leichtentritt, R. D., & Rettig, K. D. (2000). The good death: Reaching an inductive understanding. *Omega-Journal of Death and Dying,**41*(3), 221–248. 10.2190/2GLB-5YKF-4162-DJUD 10.2190/2GLB-5YKF-4162-DJUD 12557884

[CR32] Martínez Mateo, M., Cabanis, M., Stenmanns, J., & Krach, S. (2013). Essentializing the binary self: Individualism and collectivism in cultural neuroscience. *Frontiers in Human Neuroscience,**7*, 289. 10.3389/fnhum.2013.00289 23801954 10.3389/fnhum.2013.00289 PMC3689037

[CR33] McSweeney, B. (2009). Dynamic diversity: Variety and variation within countries. *Organization Studies,**30*(9), 933–957. 10.1177/0170840609338983

[CR26] Musse, I. A., Kampee-panya-withet, K., Bhagwat, M., Rabin, D., Suropada, J. S., & Danaher, P. (2017). Multifaith statement on the voluntary assisted dying bill 2017. Melbourne: Ecumenical & Interfaith Commission. Retrieved from https://www.cam1.org.au/Portals/66/Multifaith%20Statement%20on%20the%20Euthanasia%20Bill-A5.pdf

[CR34] Nathan, G. (2015). A non-essentialist model of culture: Implications of identity, agency and structure within multinational/multicultural organizations. *International Journal of Cross Cultural Management,**15*(1), 101–124. 10.1177/1470595815572171

[CR35] New South Wales Jewish Board of Deputies (2022). Communal Directory. New South Wales Jewish Board of Deputies. Retrieved from https://nswjbd.org.au/constituent-organisations/

[CR36] Osland, J. S., & Bird, A. (2000). Beyond sophisticated stereotyping: Cultural sensemaking in context. *Academy of Management Perspectives,**14*(1), 65–77. 10.5465/ame.2000.2909840

[CR37] Owens, A., & Randhawa, G. (2004). ‘It’s different from my culture; they’re very different’: Providing community-based, ‘culturally competent’palliative care for South Asian people in the UK. *Health & Social Care in the Community,**12*(5), 414–421. 10.1111/j.1365-2524.2004.00511.x 15373820 10.1111/j.1365-2524.2004.00511.x

[CR38] Oyserman, D. (2019). The essentialized self: Implications for motivation and self-regulation. *Journal of Consumer Psychology,**29*(2), 336–343. 10.1002/jcpy.1093

[CR39] Parliament of Victoria. (2016). Inquiry into end of life choices. Parliament of Victoria. Retrieved from https://www.parliament.vic.gov.au/get-involved/inquiries/inquiry-into-end-of-life-choices/

[CR40] Rabbinical Council of Victoria. (2017). RCV statement on the proposed Victorian Euthanasia bill. Rabbinical Council of Victoria. Retrieved from https://www.rcv.org.au/single-post/2017/06/13/RCV-Statement-on-the-Proposed-Victorian-Euthanasia-Bill

[CR41] Romain, J., & Carey, G. (2021). There is nothing holy about agony: Religious people and leaders support assisted dying too. *British Medical Journal*, *374*, n2094. 10.1136/bmj.n2094 34497050 10.1136/bmj.n2094

[CR42] Schweda, M., Schicktanz, S., Raz, A., & Silvers, A. (2017). Beyond cultural stereotyping: Views on end-of-life decision making among religious and secular persons in the USA, Germany, and Israel. *BMC Medical Ethics,**18*(1), 1–11. 10.1186/s12910-017-0170-4 28212642 10.1186/s12910-017-0170-4 PMC5316158

[CR44] Sen, A. (2007). *Identity and violence: The illusion of destiny*. Penguin Books India.

[CR45] Shine, R. (2019). Voluntary euthanasia legislation leaves WA’s religious communities debating doctrine and death. *Australian Broadcasting Corporation*. Retrieved 10 Aug 2019 from https://www.abc.net.au/news/2019-08-10/where-do-different-religions-stand-on-voluntary-euthanasia/11399138

[CR46] Sinclair, D. (2017). What Jewish law says about suicide and assisted dying. *The Conversation*. Retrieved 20 Dec 207 from https://theconversation.com/what-jewish-law-says-about-suicide-and-assisted-dying-88687

[CR12] Standing Committee on Legal and Social Issues. (2015). Transcript. [Parliament of Victoria Legislative Council Legal and Social Issues Committee Public hearing for the Inquiry into end of life choices] Parliament of Victoria. Retrieved 14 Oct 2015 from https://www.parliament.vic.gov.au/48e9cc/contentassets/d3a28e914f824ffdae9c23e22e6c520c/institute_for_judaism_and_civilization_-_final_-_sclsi_end-of-life_choices_14_october_2015.pdf

[CR48] Truong, M., Paradies, Y., & Priest, N. (2014). Interventions to improve cultural competency in healthcare: A systematic review of reviews. *BMC Health Services Research,**14*(1), 1–17. 10.1186/1472-6963-14-99 24589335 10.1186/1472-6963-14-99 PMC3946184

[CR49] Victorian Government Department of Health. General information about Voluntary Assisted Dying. (2019). Department of health. Retrieved 25 July 2019 from https://www.health.vic.gov.au/patient-care/general-information-about-voluntary-assisted-dying#what-is-voluntary-assisted-dying

[CR50] Voluntary Assisted Dying Act 2017 (VIC). Retrieved from https://www.legislation.vic.gov.au/in-force/acts/voluntary-assisted-dying-act-2017/006

[CR51] Wenger, N. S., & Carmel, S. (2004). Physicians’ religiosity and end-of-life care attitudes and behaviours. *International Journal of Medicine,**6*(1), 15–22. 15543435

[CR52] Zamer, J. A., & Volker, D. L. (2013). Religious leaders’ perspectives of ethical concerns at the end of life. *Journal of Hospice & Palliative Nursing,**15*(7), 396–402. 10.1097/NJH.0b013e31829cffa4

